# Supplementary material for: Multisensory flicker modulates widespread brain networks and reduces interictal epileptiform discharges
Source: Nat Commun. 2024 Apr 11;15:3156. doi: 10.1038/s41467-024-47263-y (PMC11009358; doi:10.1038/s41467-024-47263-y)
Supplement: Supplementary file 1 — Supplementary Information [file 41467_2024_47263_MOESM1_ESM.pdf]

SUPPLEMENTARY MATERIAL

for

Multisensory Flicker Modulates Widespread Brain Networks and Reduces Interictal Epileptiform Discharges in Humans

Authors

Lou T. Blanpain[1,2,3], Eric R. Cole[1,3], Emily Chen[1], James Park[1], Michael Y. Walelign[4], Robert E. Gross[1], Brian T. Cabaniss[5], Jon T. Willie[6]*, Annabelle C. Singer[2,3]*

*co-corresponding authors

SUPPLEMENTARY TABLES:

Subject	Sex	Language dominance (as determined by fMRI)
**01**	F	L
**02**	F	L
**03**	M	L
**04**	F	L
**05**	M	L
**06**	F	L
**07**	F	L
**08**	M	B
**09**	M	L
**10**	F	L
**11**	F	L
**12**	M	indeterminate
**13**	F	L
**14**	M	L
**15**	F	R
**16**	F	L
**17**	F	L
**18**	M	L
**19**	M	L

Table S1. Subject demographics

M - male; F - female; R - right; L – left; B – bilateral. Subjects included 11 participants ages 20-29, 3 participants ages 30-39, 3 participants 40-49, and 2 participants ages 50-59.

Subject	Prescribed AEDs	AEDs on day of testing	Preoperative imaging findings	Determined seizure focus (broader classification)
**01**	Levetiracetam, lamotrigine	Flicker 5.5Hz-80Hz range session: none<br>Flicker 5.5-40-80Hz session 1: lamotrigine, levetiracetam<br>Flicker 5.5-40-80Hz session 2: none	No abnormal findings	Left medial temporal (TLE)
**02**	Levetiracetam, zonisamide	None	No abnormal findings	Left basal/lateral temporal (TLE)
**03**	Lamotrigine, levetiracetam, topiramate	Lamotrigine, levetiracetam, topiramate	History of prior left medial occipital-parietal resection, possible bilateral hippocampal sclerosis.	Right temporo-occipital region (TLE)
**04**	Clobazam, lamotrigine, perampanel	Clobazam, lamotrigine	Small left frontal white matter cavernous malformation with associated developmental venous anomaly.	Left posterior frontal/ perirolandic (FLE)
**05**	Lamotrigine	Flicker 5.5-40-80Hz session: lamotrigine<br>Single-pulse session: none	Possible anterior right frontal focal cortical dysplasia.	Left mesial temporal (TLE)
**06**	Zonisamide, lamotrigine, levetiracetam	Lamotrigine, levetiracetam, zonisamide	History of left hippocampal sclerosis, expected post-operative findings of mesial temporal ablation.	Left temporal (TLE)
**07**	Clobazam, levetiracetam, phenytoin.	Flicker 5.5-40-80Hz session: none<br>Single-pulse session: levetiracetam, lorazepam	Right hemispheric atrophy, right mesial temporal sclerosis.	Right temporo-occipital region (TLE)
**08**	Lacosamide, lamotrigine	Lacosamide, lamotrigine	Expected post-operative findings of left temporal pole ablation.	Left orbitofrontal region (FLE)

**09**	Topiramate, brivaracetam, lamotrigine	Flicker 5.5-80Hz range session 1: brivaracetam (100mg), lamotrigine (300mg) Flicker 5.5-80Hz range session 2: brivaracetam (100mg), lamotrigine (100mg)	Expected postoperative findings of left amygdala-hippocampal ablation	Bilateral medial temporal (TLE)
**10**	Levetiracetam, Lamotrigine, Oxcarbazepine	Flicker 5.5-40-80Hz session: lamotrigine (125mg), levetiracetam (500mg) Flicker 5.5-80Hz range session 1: levetiracetam (250mg), lamotrigine (100mg) Flicker 5.5-80Hz range session 2: lamotrigine (50mg), levetiracetam (500mg)	Normal	Poorly localized, left hemisphere (other)
**11**	levetiracetam, lamotrigine, lorazepam, gabapentin	Flicker 5.5-40-80Hz session: lamotrigine, levetiracetam Single-pulse session: none	Question of medial left temporal cortical displasia.	Left medial temporal (TLE)
**12**	Topiramate, perampanel, lacosamide, levetiracetam	Lacosamide (150mg), levetiracetam XR (1500mg)	Possible left mesial temporal sclerosis	Multiple, bilateral fronto-temporal (other)
**13**	Levetiracetam, lamotrigine	Flicker 5.5-80Hz range session 1: lamotrigine (100mg) Flicker 5.5-80Hz range session 2: levetiracetam (250mg)	History of left temporal lobe low-grade (WHO grade 1) tumor, bilateral gray matter heterotopias, hypothalamic hamartoma, retrocerebellar cyst.	Poorly localized, multifocal; onset possibly left mesial temporal, bilateral, or multifocal (TLE)
**14**	Topiramate, phenytoin, gabapentin, clonazepam	Flicker 5.5-40-80Hz session: phenytoin Single-pulse session: none	Left frontal lobe polymicrogyria and associated closed-lip scattered schizencephaly.	Left fronto-parietal region (FLE)
**15**	Lacosamide	None	Bilateral occipital periventricular nodular heterotopia.	Right parieto-occipital region (TLE)

**16**	Eslicarbazepine, lamotrigine	Lamotrigine	Likely left hypothalamic hamartoma.	Bilateral medial temporal (TLE)
**17**	Lamotrigine, lacosamide, perampanel.	Flicker 5.5-40-80Hz session: lamotrigine Single-pulse session: lamotrigine	Expected post-operative findings of left medial temporal ablation.	Left posterior parahippocampal area (TLE)
**18**	Carbamazepine, levetiracetam	None	History of radiosurgery of left temporal lobe and frontal operculum arteriovenous malformation.	Left planum temporale, Heschl's gyrus, pars opercularis (TLE)
**19**	Levetiracetam, zonisamide, Lamotrigine.	Flicker 5.5-40-80Hz session: lamotrigine Single-pulse session: lamotrigine, levetiracetam	Possible right hippocampal atrophy.	Bilateral medial temporal (TLE)

Table S2. Epilepsy information for each subject

AED – anti-epileptic medication; TLE – temporal lobe epilepsy; FLE – frontal lobe epilepsy. In the last column, classification in parentheses (i.e., TLE vs FLE) was used to segregate analyses between the two seizure onset zone patient groups, in Figure S9.

Subject	IED rate (IED/min)	Seizure events		
		clinical	subclinical	total
**01**	40.6, 50.9, 47.4	2	0	2
**02**	25.4	1	0	1
**03**	122.7	1	+	1+
**04**	22.8	17	1	18
**05**	36.6	7	0	7
**06**	15.7	22	6	28
**07**	61.0	11	20	31
**08**	54.5	4	0	4
**09**	44.2, 51.4	3	1	4
**10**	30.9, 26.1, 68.3	5	4	9
**11**	32.6	5	0	5
**12**	19.2	21	2	23
**13**	45.1, 19.9	21	1	22
**14**	9.6	5	0	5
**15**	42.3	11	0	11
**16**	79.9	13	4	17
**17**	30.0	5	1	6
**18**	33.2	2	1	3
**19**	44.5	4	+	4+

Table S3. Intracranial monitoring activity per subject
IED – interictal epileptiform discharge; n/a – not available; + – multiple, not counted. IED rate was based on number of IEDs detected over the duration of the Flicker 5.5/40/80Hz and/or Flicker 5.5-80Hz range experimental sessions, which included periods with and without flicker stimulation.

Subject	Paradigm					
	Flicker 5.5-40-80Hz		Single-pulse		Flicker 5.5-80Hz range	
	Brightness (Lux)	Volume (dbA)	Brightness	Volume	Brightness	Volume
**01**	212	79	n/a		233	n/a
**02**	189	77	n/a		n/a	n/a
**03**	199	82	n/a		n/a	n/a
**04**	n/a		n/a		n/a	85
**05**	978	89	970	93	n/a	n/a
**06**	163	76	n/a		n/a	n/a
**07**	136	72	1029	96	n/a	n/a
**08**	1125	80	n/a		n/a	n/a
**09**	n/a		n/a		57	85
**10**	135	94	n/a		182	88
**11**	49	78	715	93	n/a	n/a
**12**	n/a		n/a		n/a	78
**13**	n/a		n/a		82	79
**14**	122	80	162	96	n/a	n/a
**15**	n/a		n/a		158	n/a
**16**	14	83	n/a		n/a	n/a
**17**	815	72	1003	74	n/a	n/a
**18**	n/a		n/a		n/a	95
**19**	880	84	1063	100	n/a	n/a
**Total subjects (total sessions)**	13 (14)		6 (6)		8 (11)	

Table S4. Paradigm and sensory stimulation amplitudes per subject

A total of 19 subjects completed one or more of three paradigms (see Figures 1B, 4B, and 5A and Methods for details). For each subject and paradigm, participation as well as brightness and volume (averaged between left and right sides of the glasses or earbuds) measured at 40Hz are indicated.

Description	Artifact	Multi-unit	Single-unit
Average waveform shapes (visual inspection) for all unit sub-clusters (can be helped by plotting of all waveforms)	Looks like artifact	Does not look like artifact	Does not look like artifact
Firing rate (per second)	<=0.05	>0.05	>0.05
Fraction of inter-event intervals <3ms	>=0.1	>=0.05	<0.05
Narrowing of main peak of waveforms' density plot (visual inspection)	Narrowing	Narrowing	No narrowing
Local peaks (positive or negative) following main peak of average waveforms (visual inspection) for all sub-clusters	>=4	3	<3
Distribution of the event amplitudes (visual inspection)	-	Multimodal	Unimodal

Table S5: Criteria for classifying a group as artifact, multi-unit, or single-unit.

SUPPLEMENTARY FIGURES:

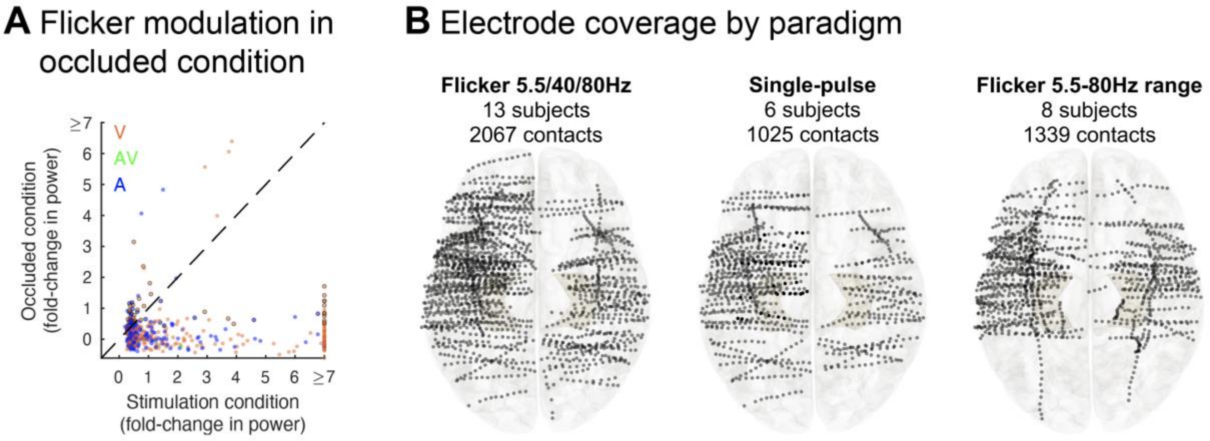

Figure S1. Relative occluded condition modulation and electrode coverage by paradigm

(A) Out of contacts that showed significant flicker modulation to 40Hz visual, auditory or audiovisual flicker in the Flicker 5.5/40/80Hz paradigm, we represented the corresponding fold-change in power (capped at 7) at the frequency of stimulation for the relative occluded condition versus the non-occluded condition. Each dot indicates a contact's responses for a given modality in one recording session with orange, green and blue dots representing visual (V), audio-visual (AV), or auditory (A) stimulus conditions, respectively, and dots circled in black representing results that are significant in the relative occluded condition. Significant modulation in the occluded condition may suggest our occluded condition is not completely successful in occluding sensory stimuli from the subject's visual and auditory systems. For rare cases where we observed a clear peak at the frequency of stimulation in the PSD for the occluded condition, in the majority of those cases the peak was smaller than in the non-occluded condition, which suggests it may be true sensory modulation from imperfect occlusion of the sensory stimuli, rather than noise from the flicker device. Overall, the vast majority of contacts showed stronger modulation in the non-occluded condition. This indicates low noise levels using our experimental and preprocessing methods.

(B) Number of subjects and number of contacts (above), as well as approximate location of each contact (represented by dots) across patients on Montreal Neurological Institute (MNI) normalized 3D brain (top view) for each of the three paradigms tested (see Figure 1B, 4B and 5A for details). Note: number of channels included in an analysis may vary by 1-3 channels, depending on which channels were too noisy for that session; here we report the number of channels from one of the sessions for each subject.

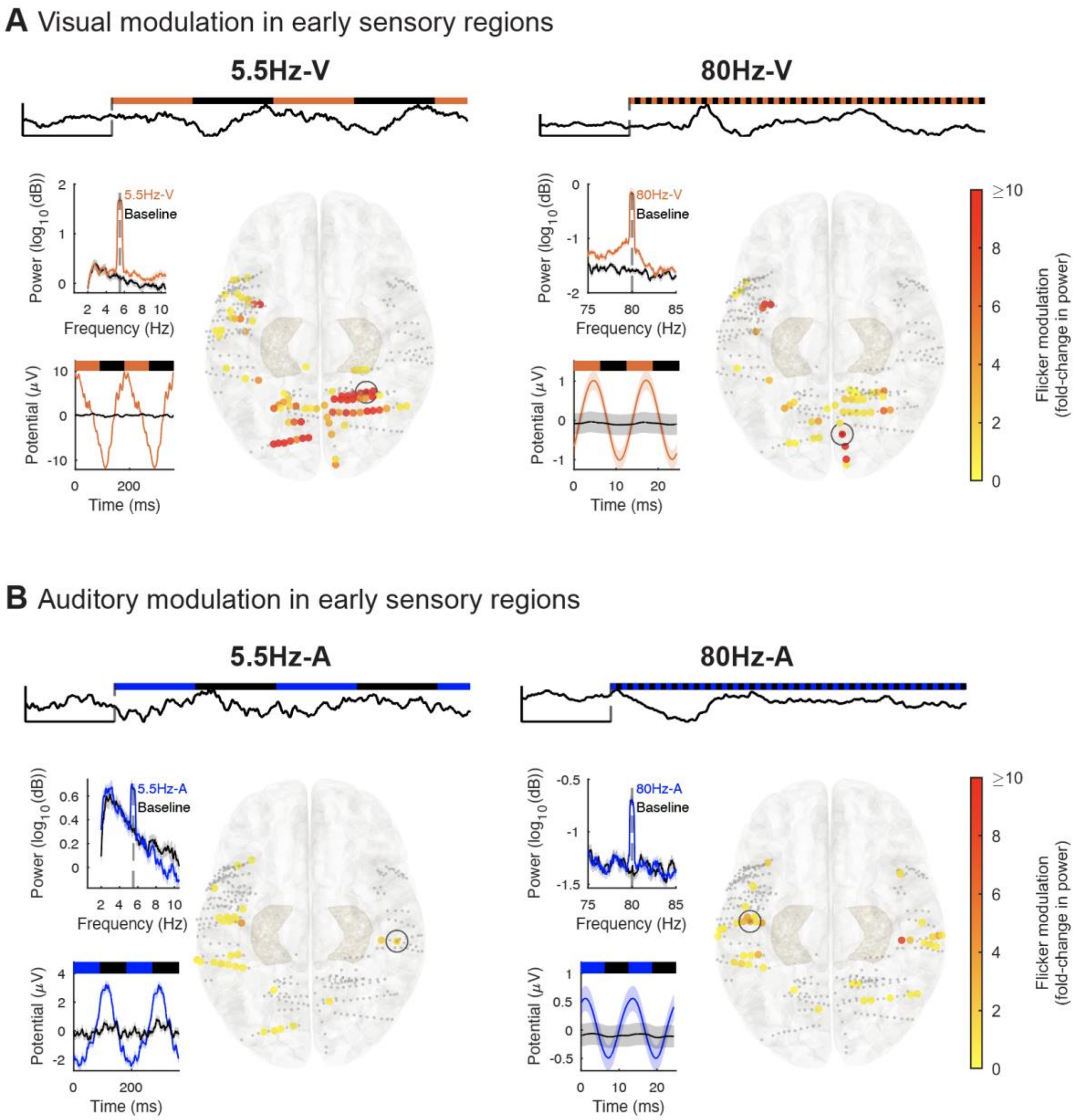

<u>*Figure S2.Responses to 5.5Hz and 80Hz visual and auditory stimulations in early sensory areas*</u>

(A) Response to 5.5Hz-visual flicker (left) and 80Hz-visual flicker (right) in early visual and auditory areas (n = 337 channels across 12 sessions and subjects). For each panel, the 3D brain plot represents contacts located in early visual and auditory regions, across patients, on normalized Montreal Neurological Institute (MNI) brain (top view), with color indicating the degree of modulation at the frequency of stimulation. Top represents the minimally preprocessed LFP trace from contact highlighted with black circle in 3D brain plot, left top represents average power spectral density of the response to 5.5Hz-visual stimulation (orange) versus baseline (black); bottom left represents responses averaged over 2 cycles of the stimulus. Dark line is mean and shaded area is standard error of the mean.

(B) Same as (A) but for responses to 5.5Hz and 80Hz auditory stimulations (blue) and baseline (black).

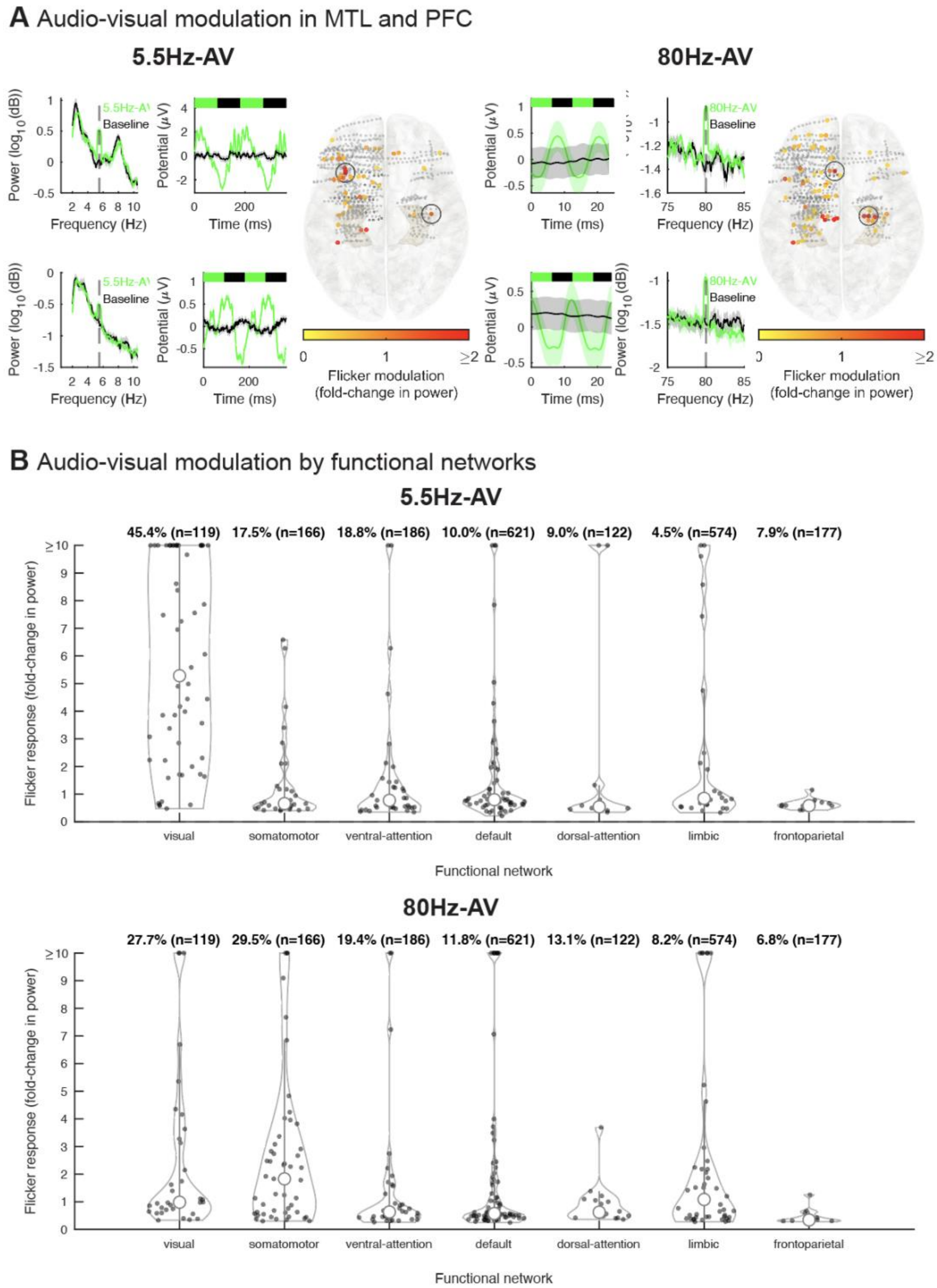

<u>Figure S3. Response to 5.5Hz and 80Hz audio-visual stimulation in higher cognitive regions</u>

(A) Left: two example contacts (one contact per row) and their responses to 5.5Hz-audiovisual flicker (green), represented in the frequency (first column) and time (second column) domains, compared to baseline (black); summary of the response to 5.5Hz-audiovisual flicker is represented on 3D normalized Montreal Neurological Institute (MNI) brain (top view), in the medial temporal

lobe and prefrontal cortex (third column; total of 793 contacts across 13 sessions and subjects). Contacts highlighted with a black circle are ones which responses are represented on the left. Right: same as left but illustrating the response to 80Hz-audiovisual stimulation. Dark line is mean and shaded area is standard error of the mean.

(B) Top: distribution of the amplitudes of the response to 5.5Hz-audiovisual stimulation by functional network (total of n = 1,965 contacts across 13 sessions and subjects). Numbers at the top indicate the percent showing a significant response in each functional network, and total number of contacts in each network. Bottom: same at top but illustrating the response to 80Hz-audiovisual stimulation. Each dot is one contact.

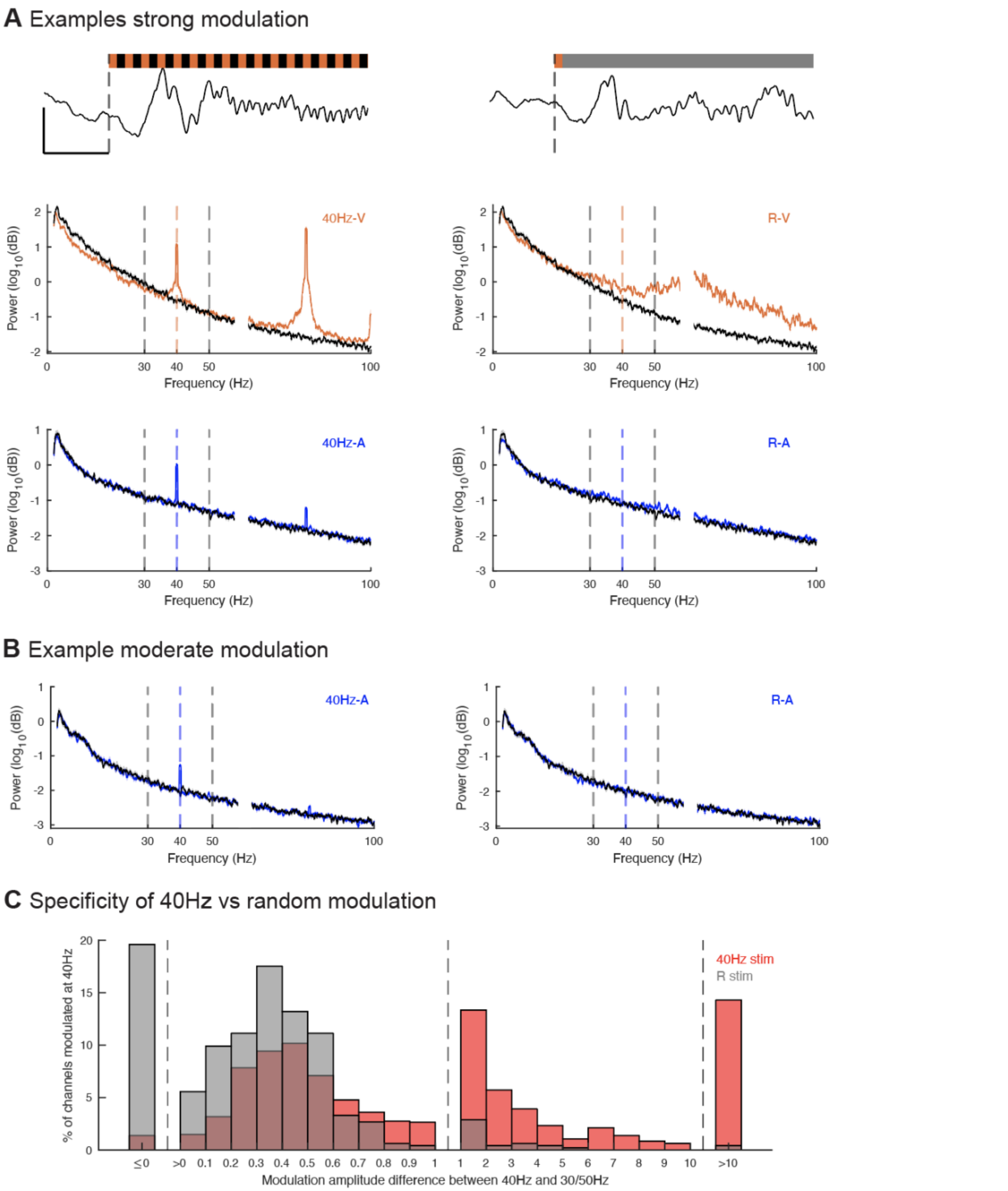

Figure S4. Distinct responses to periodic versus random sensory flicker

To contrast the responses to periodic versus random flicker stimulation, we compared the specificity of modulation at the frequency of stimulation of 40Hz versus random conditions.

(A) Example contacts with strong sensory responses. Top: example start of evoked potential averaged across trials for one contact, in the 40Hz-visual stimulation condition (left) and random visual stimulation condition (right); below: corresponding power spectral density plots; bottom:

power spectral density plots for the response of another contact to 40Hz-auditory stimulation (left) and random-auditory stimulation (right). In power density plots, orange, blue and black represent visual, auditory stimulation and baseline conditions, respectively. For both contacts, we observe a strong, narrow-frequency band response to periodic stimulation at the frequency of stimulation, and a broader frequency response in the random flicker condition.
(B) Example of a contact with mild response to 40Hz-auditory flicker, and minimal to no response to random auditory flicker; format as in (A).
(C) The distribution of the difference between fold-change increase in power at 40Hz (versus baseline) and the average fold-change at 30Hz and 50Hz (versus baseline), in the 40Hz stimulation condition (red) compared to random (grey) (n = 14 sessions across 13 subjects). Only data from contacts showing significant increase in power at 40Hz in the periodic or random conditions, respectively, were included. To facilitate visualization, the x-axis was subdivided into values <= 0, between 0 and 1, between 1 and 10, and >10. The random condition is mostly distributed in the range below 1, while the periodic condition is mostly distributed in the range above 1, indicating a non-specific or minimal modulation in the random condition, compared to a frequency-specific modulation in the periodic condition.

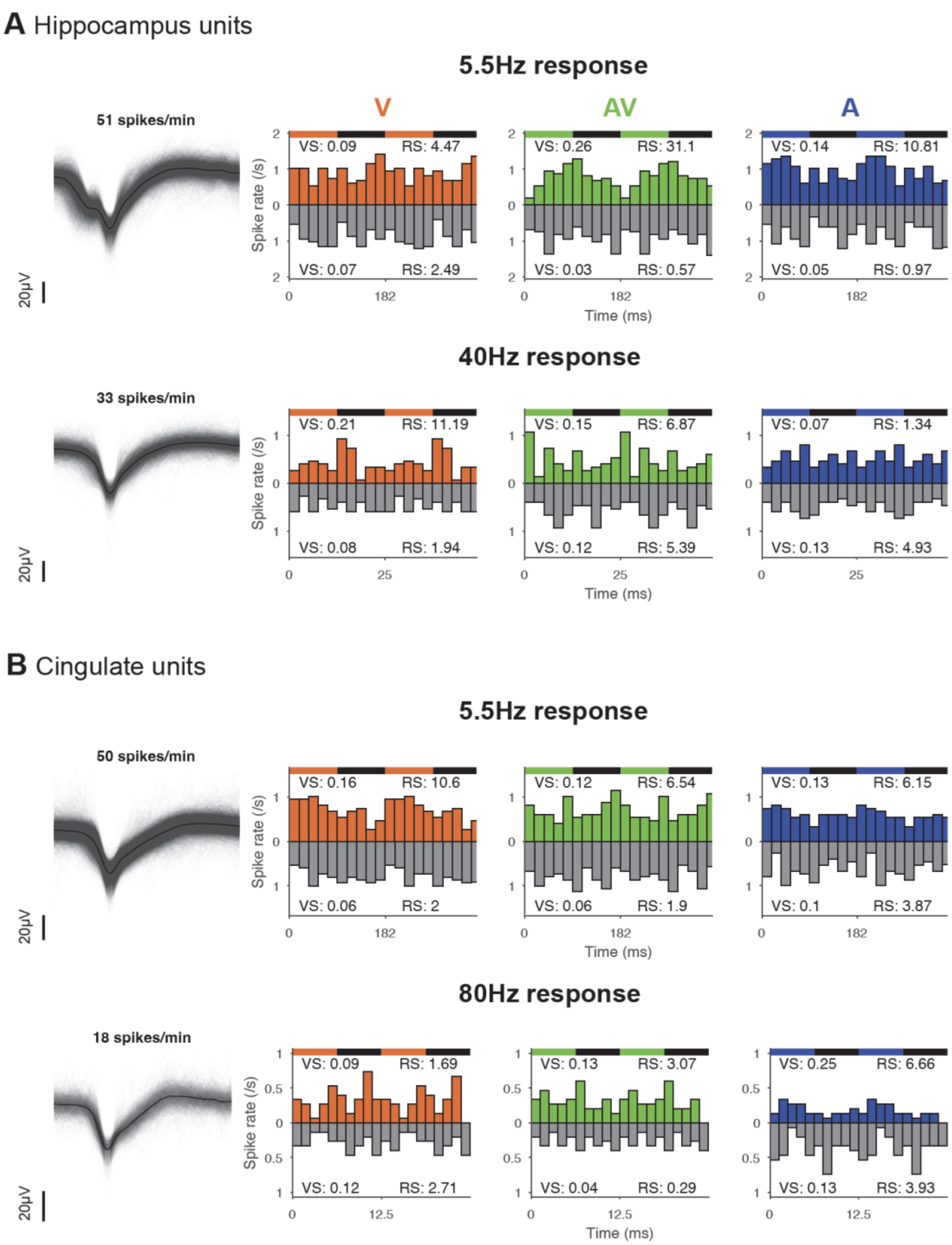

Figure S5. Flicker modulates neurons' spiking activity in the human hippocampus and cingulate

To study neuron spiking activity, four subjects were also implanted with depth electrodes containing microwires (Figure 1A) that terminated in the hippocampus and cingulate, and a total of 25 units (13 single units, 12 multi-units) were isolated. Out of them, 21 units (7 single units and 3 multi-units in the hippocampus, 5 single units and 6 multi-units in the cingulate) had a spike rate that was high enough to assess modulation (see Methods).

(A) Example single neuron waveforms (left; solid line represents average waveform, transparent lines represent individual waveforms), with peristimulus-time histograms (right) averaged over 2 cycles of the stimulus, illustrating from left to right response to 5.5Hz visual (V, orange), audio-visual (AV, green), and auditory (A, blue) stimulation (colored bars) versus random condition (grey inverted bars). Vector strength (VS) and Rayleigh statistics (RS) for each condition are indicated on the top and bottom of the plot. We see a higher average firing rate at a given phase (between ON/OFF) of the stimulus for the AV condition, showing that this unit is more strongly modulated by 5.5Hz-AV flicker. Bottom: same illustration for a hippocampal multi-unit, in response to 40Hz flicker. This unit seems to be more strongly modulated in the visual modality (also preferred the ON/OFF phase of stimulus).

(B) Same as (A) for cingulate units, showing response to 5.5Hz flicker (top) and 80Hz flicker (bottom). Top shows single neuron with stronger modulation to 5.5Hz-V stimulation (higher average firing rate at the early ON phase of stimulus), while bottom shows multi-unit with stronger modulation to 80Hz-A stimulation (preferred middle of ON phase of stimulus).

This constitutes preliminary evidence that some neural units in hippocampus and cingulate respond to flicker with higher average firing rates at particular phases of the stimulus.

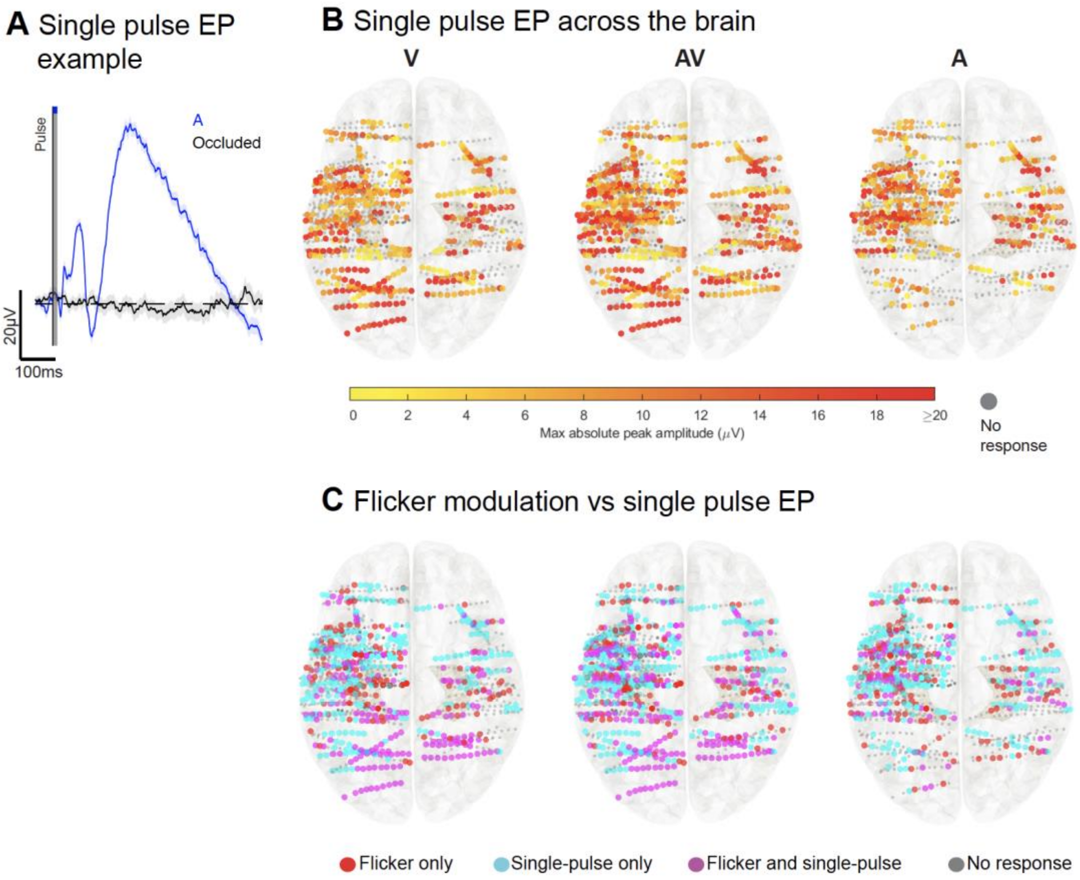

Figure S6. Single-pulse evoked potential across the brain

(A) Example evoked potential (EP), averaged across 200 trials, to auditory (A, blue) versus relative occluded audio-visual (black) pulses, in the primary auditory cortex; solid line represents the mean, shaded area represents standard error of the mean. As expected, we see a rapid (first peak ~20ms), large (up to ~50μV) response to auditory pulse compared to the relative occluded condition.

(B) Approximate location and associated single pulse EP amplitudes of contacts (illustrated with dots) represented on 3D Montreal Neurological Institute (MNI) normalized brain (top view), for visual (V, left), audio-visual (AV, center) and auditory (A, right) modalities, capped at 20μV (total of n=1025 contacts across 6 sessions and subjects). Smaller grey dots represent non-significant single pulse EP responses, while large dots represent significant responses, with maximal absolute peak from 0μV (yellow) to 20μV or more (red). There were 97, 148 and 71 contacts with amplitude values higher than 20μV respectively in the visual, audio-visual, and auditory modalities. As expected, we see a strong response to conditions involving the visual modality in the occipital region, but also in the parietal, temporal, and prefrontal regions. Strong responses to the auditory condition were observed in the temporal region, but also the prefrontal region.

(C) Responses to single-pulse versus flicker: approximate location of contacts (represented by dots) and their responses to visual (left), audiovisual (middle) and auditory (right) modalities, represented on 3D MNI brain (top view; total of n = 1021 contacts across 12 sessions with 6 sessions of single pulse paradigm and 6 sessions of flicker paradigm, in 6 subjects). Contacts show

responses to flicker-only (red), single pulse-only (cyan), both flicker and single pulses (purple), or no response (grey).

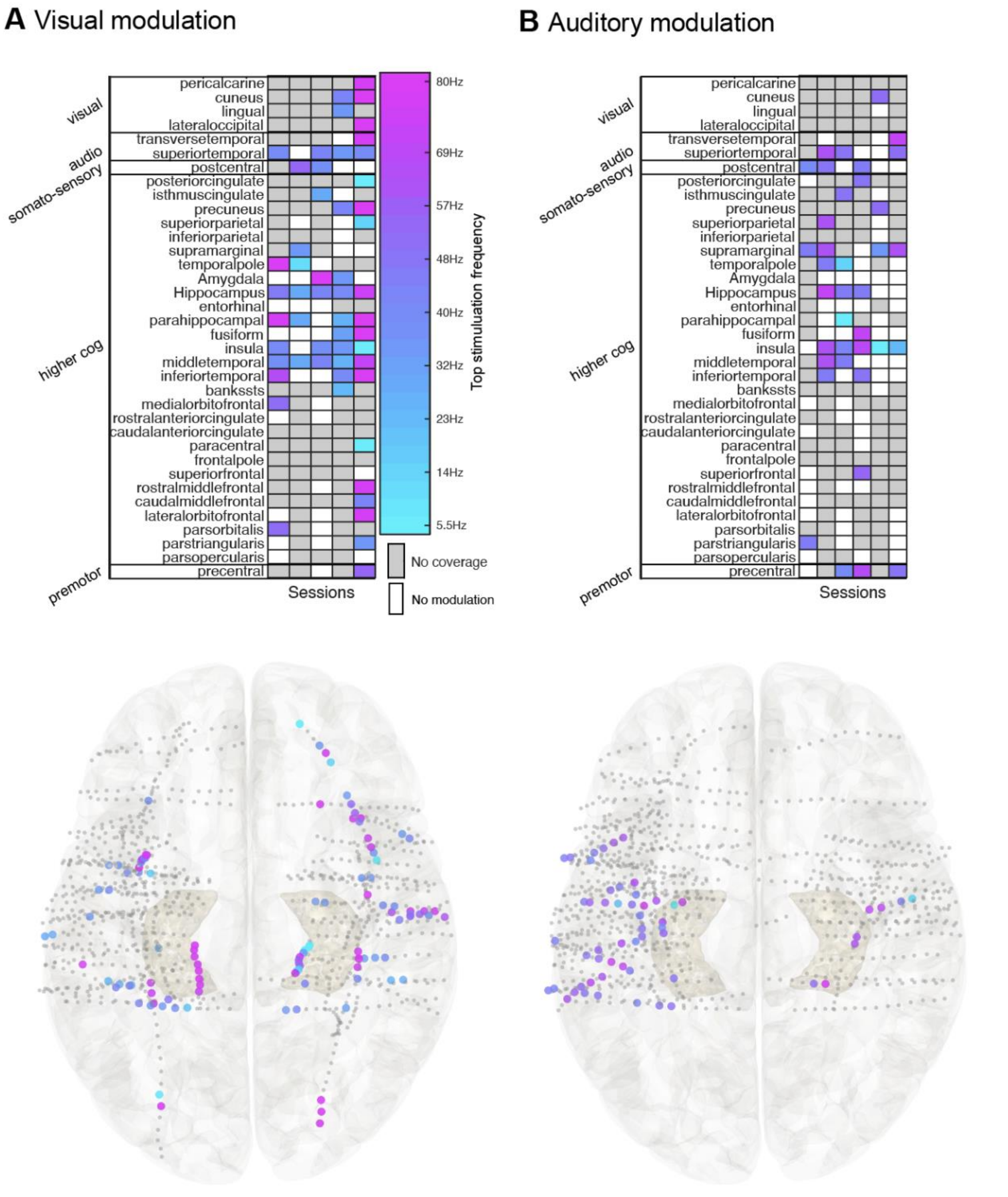

<u>Figure S7. Preferred stimulation frequency by brain region and modality</u>

(A) *Top: representation of the stimulation frequency leading to maximal fold-change in power in each respective brain region, in the case of visual stimulation (n = 943 contacts, 5 sessions across 5 subjects). Only contacts showing significant fold-change in power to more than six of the stimulation frequencies tested, were included in the analysis. Moreover, when multiple of such*

channels were located to a given region, the channel responding to the highest number of frequencies, was picked in order to determine top stimulation frequency for that region. Light blue to purple colormap indicates preference for increasing frequencies of stimulation (from 5.5-80Hz); grey indicates no coverage in that region; white indicates there was no significant modulation to more than 6 stimulation frequencies in that region. Bottom: 3D representation of contacts (one dot per contact) across normalized Montreal Neurological Institute (MNI) brain from all subjects, color-coded by their preferred frequency of stimulation. Details of the analysis and colormap similar to top.

(B) Same as (A) but for sessions involving auditory stimulation (n = 959 contacts, 6 sessions across 6 subjects).

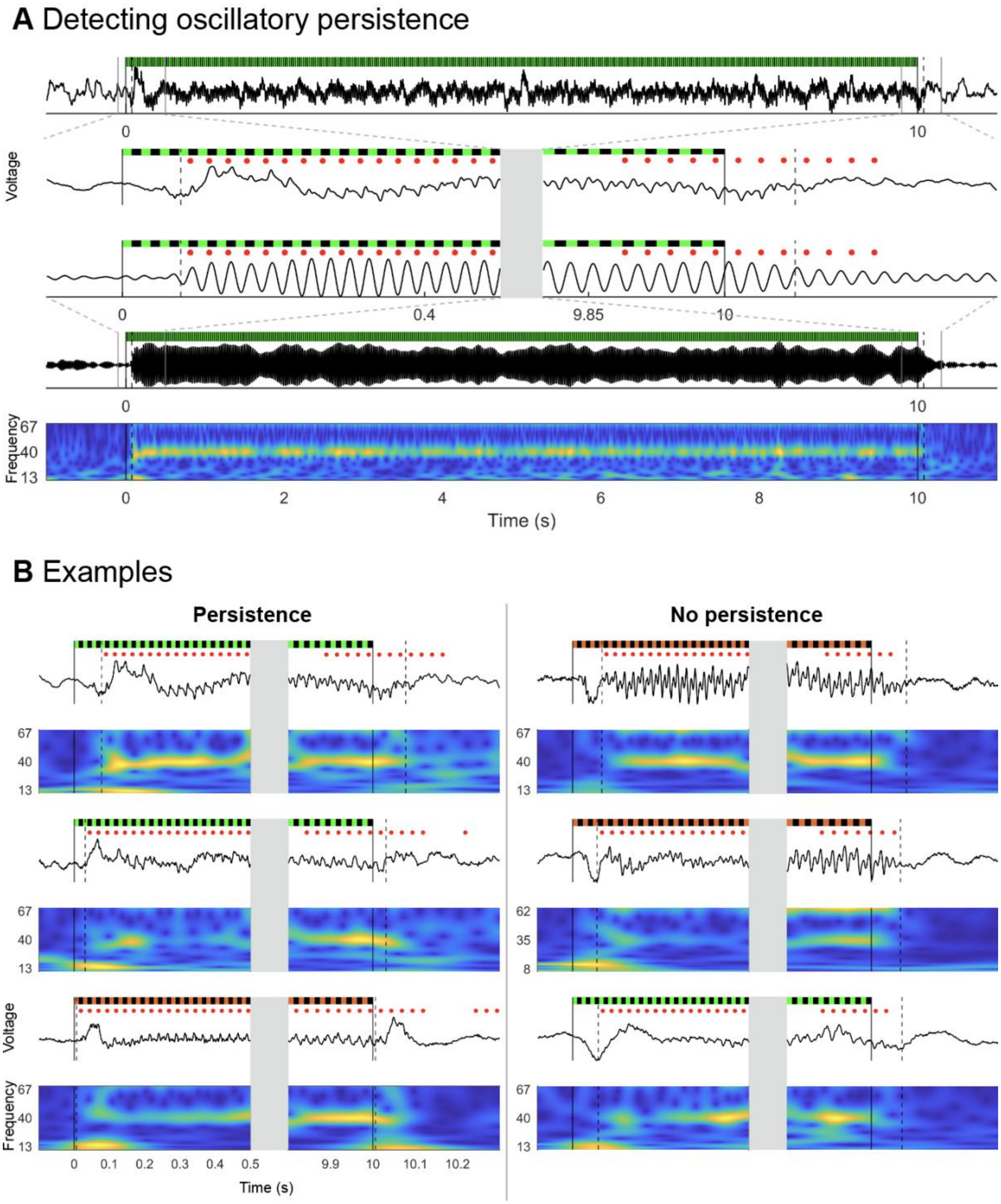

<u>*Figure S8. Evidence for persistent oscillatory response to sensory flicker*</u>

(A) Example detection of persistent oscillatory activity, using methods from Pesnot et al 2021[1]*- top row: average evoked response to 10 seconds of 40Hz-audiovisual flicker in one example contact; second row: zoom-in on the first ~500ms and last ~200ms of the averaged evoked potential; third row: corresponding zoom-in of the averaged evoked potential band-pass filtered at the frequency of stimulation (40Hz) +/-0.5Hz; fourth row: whole trial duration band-pass*

filtered (at frequency of stimulation +/-0.5Hz) signal; 5th row: time-frequency plot of the averaged evoked potential. Green indicates the 40Hz-audiovisual condition, red dots indicate cycles of the sensory response with significant oscillatory activity around the frequency of stimulation; solid vertical lines – start and end of 10s trial, dashed vertical lines – detected start and expected end of oscillatory response due to sensory processing delay. In this example contact, we observed persistent oscillatory activity, of about 3 cycles, beyond where we would expect the oscillatory response to stop.

(B) Examples of persistence (left, 1 example per 2 rows), and no persistence (right) of oscillatory response after sensory flicker offset; first row represents averaged evoked potential, second row represents its time-frequency plot (with middle frequency indicated on the Y-axis being the frequency of stimulation); the first ~500ms and last ~200ms of the response are represented separated by a grey rectangle; green indicates audiovisual stimulation, orange indicates visual stimulation.

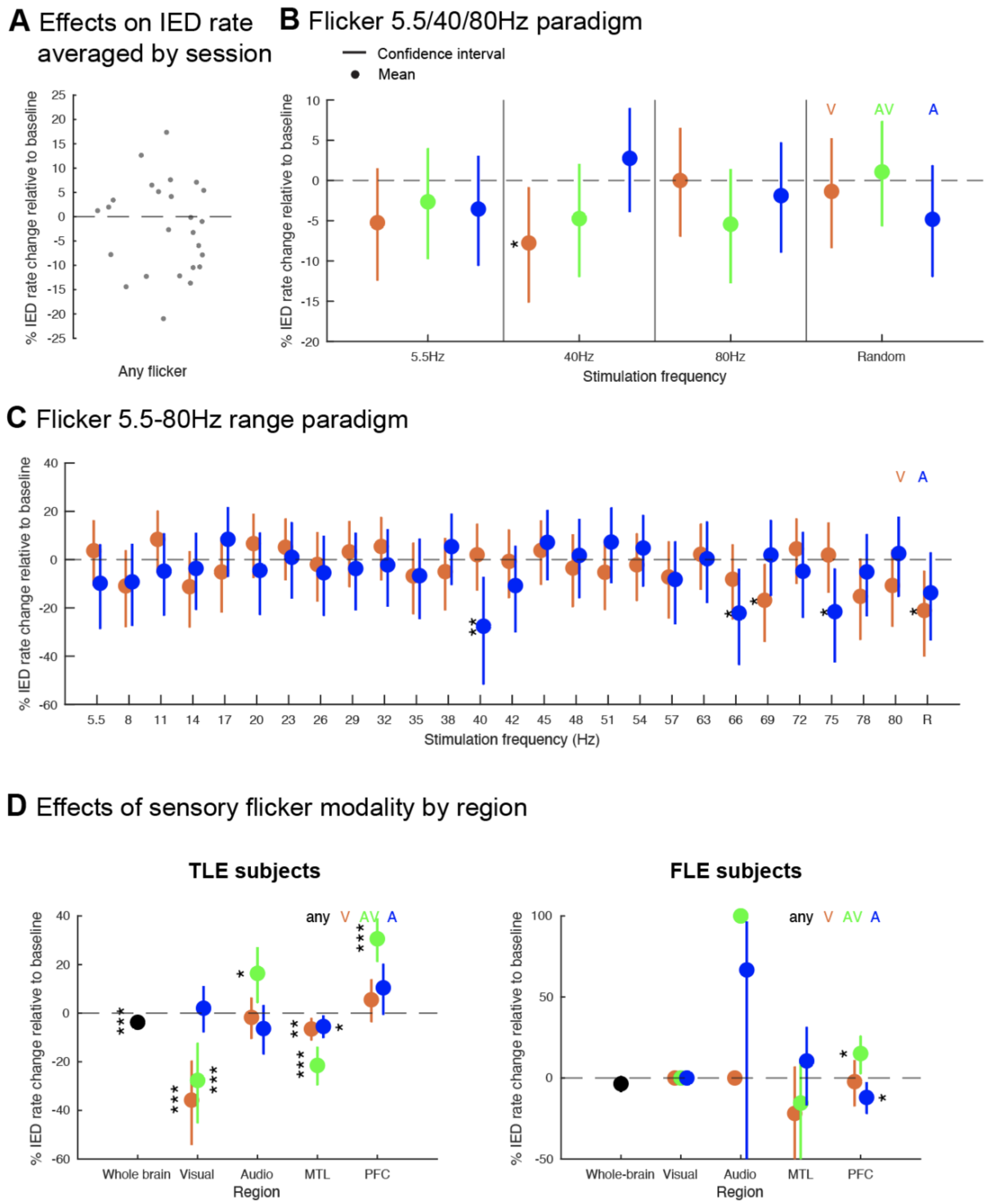

Figure S9. Effects of sensory flicker on IED rate by flicker condition

(A) Overall effect of any sensory flicker stimulation on the raw IED rate, averaged per session (n = 25 sessions in 19 subjects); each dot represents the percent change between mean baseline IED rate and mean flicker IED rate per session summing together the IED counts over all recorded

channels. Note that these data are raw percent changes from baseline and, unlike the percent change results from the Poisson generalized linear effects models (shown in the Fig 6 and Figures S9B, C, D), these values do not account for the discrete-valued nature of IEDs, effects of sparse IED occurrence, or patient heterogeneity (see Methods).

*(B) Effect of sensory flicker stimulation on IED rate by condition, for the Flicker 5.5/40/80Hz paradigm across the brain. Means are represented with dots, confidence intervals with vertical bars; visual or V conditions are in orange, audiovisual or AV in green, auditory or A in blue. Overall, we did not observe a significant effect of any single flicker condition on IED rate across the whole brain, except for a significant decrease in the 40Hz-V condition (n = 14 sessions across 13 subjects). Poisson generalized linear mixed effects model for all statistical comparisons, * p-value<0.05, not corrected for multiple comparisons.*

*(C) Same as (A) for the 5.5-80Hz paradigm; Overall, we did not observe a significant effect of any flicker condition on IED rate, except for a significant decrease at 40Hz-A, 66Hz-A, 69Hz-V, 75Hz-A and R-V (n = 11 sessions across 8 subjects). Poisson generalized linear mixed effects model for all statistical comparisons, * p-value<0.05, not corrected for multiple comparisons.*

(D) Effect of sensory flicker stimulation on IED rate for subjects with temporal lobe (TLE, left; n = 18 sessions in 14 subjects) or frontal lobe (FLE, right; n = 3 sessions in 3 subjects) epilepsy or seizure onset zone (SOZ). Means are represented with dots, confidence intervals with vertical bars; visual or V conditions are in orange, audiovisual or AV in green, auditory or A in blue. Overall, we found significant decreases in IED rate in the MTL of TLE patients with visual, audiovisual and auditory stimulation and a significant increase in some regions outside the SOZ general region. In frontal SOZ subjects, we found auditory flicker decreased the IED rate in PFC while audiovisual flicker increased the IED rate with no significant differences in other circuits examined. Poisson generalized linear mixed effects model for all statistical comparisons, * p-value<0.05, ** p-value<0.01, *** p-value<0.001, not corrected for multiple comparisons.
