# Supplementary material for: Multisensory flicker modulates widespread brain networks and reduces interictal epileptiform discharges
Source: Nat Commun. 2024 Apr 11;15:3156. doi: 10.1038/s41467-024-47263-y (PMC11009358; doi:10.1038/s41467-024-47263-y)
Supplement: Supplementary file 3 — Reporting Summary [file 41467_2024_47263_MOESM3_ESM.pdf]

nature portfolio

Corresponding author(s): Annabelle Singer

Last updated by author(s): Mar 4, 2024

Reporting Summary

Nature Portfolio wishes to improve the reproducibility of the work that we publish. This form provides structure for consistency and transparency in reporting. For further information on Nature Portfolio policies, see our Editorial Policies and the Editorial Policy Checklist.

Statistics

For all statistical analyses, confirm that the following items are present in the figure legend, table legend, main text, or Methods section.

n/a	Confirmed	
☐	☒	The exact sample size (*n*) for each experimental group/condition, given as a discrete number and unit of measurement
☐	☒	A statement on whether measurements were taken from distinct samples or whether the same sample was measured repeatedly
☐	☒	The statistical test(s) used AND whether they are one- or two-sided<br>*Only common tests should be described solely by name; describe more complex techniques in the Methods section.*
☐	☒	A description of all covariates tested
☐	☒	A description of any assumptions or corrections, such as tests of normality and adjustment for multiple comparisons
☐	☒	A full description of the statistical parameters including central tendency (e.g. means) or other basic estimates (e.g. regression coefficient) AND variation (e.g. standard deviation) or associated estimates of uncertainty (e.g. confidence intervals)
☐	☒	For null hypothesis testing, the test statistic (e.g. *F*, *t*, *r*) with confidence intervals, effect sizes, degrees of freedom and *P* value noted<br>*Give P values as exact values whenever suitable.*
☒	☐	For Bayesian analysis, information on the choice of priors and Markov chain Monte Carlo settings
☒	☐	For hierarchical and complex designs, identification of the appropriate level for tests and full reporting of outcomes
☒	☐	Estimates of effect sizes (e.g. Cohen's *d*, Pearson's *r*), indicating how they were calculated

Our web collection on statistics for biologists contains articles on many of the points above.

Software and code

Policy information about availability of computer code

Data collection

Local field potentials measured with macro-contacts were recorded on the clinical monitoring system (XLTEK EMU 128FS; Natus Medical) and associated Natus Neuroworks software, typically at a rate of 2,048Hz (range 1,024-16,384Hz).
Microwires were recorded using the Blackrock NeuroPort system (Blackrock Microsystems, UTSW) and associated Central Software Suite at a rate of 30,000Hz using a dedicated microwire as physical reference.
Customized software ran in MATLAB 2019b controlled stimulus presentation, including the creation of analog sensory signals and pulses synching the EEG recordings, and control of their timing (a version of the source code is available at https://github.com/singerlabgt/Behavioral_FlickerMasterTask). These signals were produced using a digital acquisition board (USB-6212 multifunction I/O device, National Instruments), which sent analog signals to a customized circuit. Opaque glasses containing LEDs (Mind Alive Inc.) administered visual stimuli, and earbuds (SONY MDR-EX15LP) presented auditory stimuli.
At the end of each session, we measured the brightness and volume of the device at 40Hz audiovisual stimulation, using a luxmeter (TRACEABLE Dual-Range Meter) and decibel meter (BAFX Products BAFX3608).

Data analysis

Custom code is available on GitHub. Code used for analysis of the minimally preprocessed data is available on GitHub at https://github.com/singerlabgt/MultisensoryFlickerHumanIntracranial. A version of the code used for generating stimulation paradigms is available on GitHub at https://github.com/singerlabgt/Behavioral_FlickerMasterTask. Details regarding references for source code and software are also found in the Methods section.

Localization of electrode contacts within the brain:
We identified and labelled electrodes on the post-operative CT using the voxTool software (https://github.com/pennmem/voxTool), then co-registered all imaging to pre-operative T1 MRI using ri.gid transformation with the Advanced Normalization Tools package (ANTs; stnava.github.io/ANTs/; Avants et al. 2011). We calculated electrode coordinates in different imaging spaces using co-registration output and

custom MATLAB scripts that incorporated a function from Lead-DBS (lead-dbs.org; Horn and Kühn 2015). Pre-operative T1 MRI was parcellated and segmented using the FreeSurfer toolbox (https://surfer.nmr.mgh.harvard.edu/; Fischl 2012). Where appropriate, here and in other preprocessing steps or analyses, we used GNU Parallel to process data in parallel (https://www.gnu.org/software/parallel/; Tange). Electrodes were anatomically labelled using FreeSurfer outputs and custom scripts.
We extracted normalized electrode locations into MNI space, through rigid, affine then symmetric image normalization (SyN) coregistration of pre-operative T1 MRI to T1 MNI MRI (ICBM152 2009b Nonlinear Asymmetric; Fonov et al. 2011, Fonov et al. 2009).

Data preprocessing and analysis:
Most analyses were run in MATLAB 2019b, using custom scripts in combination with Fieldtrip (https://www.fieldtriptoolbox.org/; Oostenveld et al. 2011) and Chronux (http://chronux.org/) toolboxes. For circular statistics, we used the Circular Statistics Toolbox (Berens 2009). For detection of endogenous oscillations, we used the FOOOF toolbox (https://github.com/fooof-tools/fooof; Donoghue 2020). Figures were produced in part using code from the export_fig toolbox (https://github.com/altmany/export_fig). Violin plots were produced using code from the Violinplot-Matlab toolbox (https://github.com/bastibe/Violinplot-Matlab; Bechtold 2016). Spikes were extracted and clustered using the Combinato Python-based software (https://github.com/jniediek/combinato; Niediek et al. 2016). For interictal epileptiform discharge (IED) detection, we used a previously validated automated IED detection algorithm (https://github.com/ecoglab/aied; Horak et al. 2015; Quon et al. 2022).

For manuscripts utilizing custom algorithms or software that are central to the research but not yet described in published literature, software must be made available to editors and reviewers. We strongly encourage code deposition in a community repository (e.g. GitHub). See the Nature Portfolio guidelines for submitting code & software for further information.

Data

Policy information about availability of data

All manuscripts must include a data availability statement. This statement should provide the following information, where applicable:

- Accession codes, unique identifiers, or web links for publicly available datasets
- A description of any restrictions on data availability
- For clinical datasets or third party data, please ensure that the statement adheres to our policy

Source data for all figures are provided with this paper. Minimally processed neurophysiological data, stimulation information, and electrode locations generated in this study have been deposited in the Data Archive for the Brain Initiative (DABI, https://dabi.loni.usc.edu) under project code BM2ZIVWKBFH8 and identifier https://doi.org/10.18120/4bfr-1x58. MRI imaging data are protected and are not available due to data privacy. Individual de-identified data is shared in supplementary tables (sex, language dominance, anti-epileptic medication, preoperative imaging findings, determined seizure focus, IED rate, and seizure events) and in the DABI database (neurophysiological data, stimulation exposure, and electrode placement). Age is provided in aggregate in the supplement to protect subject privacy. This data is available for research purposes.

Research involving human participants, their data, or biological material

Policy information about studies with human participants or human data. See also policy information about sex, gender (identity/presentation), and sexual orientation and race, ethnicity and racism.

Reporting on sex and gender	Subjects were recruited regardless of sex (female vs male) indicated on the patient clinical chart. A total of 11 females and 8 males recruited completed one or more of the paradigms run in this study. Sex and gender were not considered in the design and analyses of the study, for two main reasons: 1) We did not anticipate the neurophysiological effects of sensory stimulation to differ between females and males and 2) recruited subjects are rare, due to the small number of intracranial EEG patients seen by any institution in a given time period, so we minimized parcellation of the data into smaller groups.
Reporting on race, ethnicity, or other socially relevant groupings	We did not use categorizations of race, ethnicity, or other socially constructed categorization variables in this manuscript.
Population characteristics	See Supplementary Table 1 for population characteristics. Our study design mostly involved within subject and experimental session comparison of the neurophysiological effects of sensory stimulation, thus minimizing the need to account for potential candidate covariates such as anti-epileptic drugs given by clinicians on the day of testing, or seizure activity during the patient's stay.
Recruitment	We recruited treatment-resistant epilepsy patients undergoing pre-surgical intracranial seizure monitoring. Participants were approached by study staff before or while in the epilepsy monitoring unit and asked if they would be interested in participating in research. Recruitment criteria included: age over 18, fluent in English, able to understand and give verbal and written consent to the study procedures and associated risks, not suspected to be susceptible to photic-induced seizures or psychogenic non-epileptic seizures (PNES) triggered by sensory stimulation, did not show abnormal EEG activity if tested with clinical photic stimulation. These recruitment criteria may have biased our results on the effects of sensory flicker on interictal epileptiform discharges (IEDs), where we may have inherently actively selected for patients who would not present with IEDs or other abnormal activity in response to photic stimulation. Moreover, our patient population, constituted vastly of focal-onset seizure patients, typically is at low risk for seizures or abnormal EEG activity in response to given types of photic stimulation, which may have further biased our results. However this bias is mitigated by two considerations: 1) The reason behind excluding such patients who are at risk for photic-induced seizures or abnormal activity was to minimize risk of the research to this patient population, 2) Our overall goal was to assess whether sensory flicker may have positive effect on pathological neurological activity in this patient population. We feel that despite the noted potential bias, results still hold value as proof of concept to extend this potential intervention to other patient populations for therapeutic gains.
Ethics oversight	The Emory Institutional Review Board.

Note that full information on the approval of the study protocol must also be provided in the manuscript.

Field-specific reporting

Please select the one below that is the best fit for your research. If you are not sure, read the appropriate sections before making your selection.

☒ Life sciences ☐ Behavioural & social sciences ☐ Ecological, evolutionary & environmental sciences

For a reference copy of the document with all sections, see nature.com/documents/nr-reporting-summary-flat.pdf

Life sciences study design

All studies must disclose on these points even when the disclosure is negative.

Sample size	Based on prior studies, we anticipated that 5 to 10 subjects per group would be sufficient to test our hypotheses because our study design used within subject comparisons. For the Flicker 5.5/40/80Hz paradigm, subject sample size (originally aimed around 4-5) and trial sample size (15) per stimulation condition were educated by previous published data regarding sensory flicker modulation in rodents. We increased the number of subjects recruited partly based on aggregated electrode coverage across the brain, and the need to collect single neuron data in a subset of subjects. For the Flicker 5.5-80Hz range paradigm, based on data obtained from the Flicker 5.5/40/80Hz paradigm, we estimated that 5 subjects and 10 trials per condition would be sufficient to test our hypotheses. Number of patients was again partly based on aggregated electrode coverage across the brain. For the Single-pulse paradigm, we aimed for a number of trials (200) in the order of magnitude of what is used to study averaged sensory evoked potentials with scalp EEG. Based on data obtained from the Flicker 5.5/40/80Hz paradigm, we estimated that 5 subjects would be sufficient to test our hypotheses. Number of patients was again partly based on aggregated electrode coverage across the brain.
Data exclusions	In cases where subjects completed less than 75% of the trials, we did not include the data in the study, as it was considered that too few trials were available to infer meaningful conclusions about the neurophysiological effects of sensory stimulation in those patients.<br>We ran the Flicker 5.5/40/80Hz paradigm twice in one subject, and results from the session leading to the smallest number of contacts with significant modulation in the 40Hz audiovisual condition, were excluded from flicker modulation results. This session was included in the analysis on the effects of sensory flicker on interictal epileptiform discharges (IEDs).
Replication	We originally collected data from the Flicker 5.5/40/80Hz paradigm in a small sample size (5) and observed flicker modulation. We then collected additional data in more subjects to ensure the original findings were replicable. Ultimately we tested the effects of flicker in 19 subjects with two different stimulation paradigms. All replications were successful.
Randomization	This study's comparisons across conditions were performed within subjects, and each subject was exposed to all conditions within a single session for a given experimental paradigm.<br>For the Flicker 5.5/40/80Hz paradigm, trials were pseudo-randomized (with no given modality or frequency repeated more than three times in a row) to control for order effects and minimize habituation to a given stimulus. Each stimulation trial was followed by 10 seconds of no stimulation, i.e., a baseline trial.<br>For the Single-pulse paradigm, trials from each modality were presented in a pseudorandomized manner (no given modality repeated more than three times in a row).<br>For the Flicker 5.5-80Hz range paradigm, trials were pseudo-randomized (no given condition repeated more than three times in a row, with attempt to spread all conditions across the experiment duration) and separated by an intertrial interval randomized between 2-2.5s. In most cases, only one modality of stimulation (visual or auditory) was administered for a given subject. In those cases, the modality was typically picked based on individual subject’s electrode coverage, i.e., visual modality was selected in subjects with electrodes in areas predicted to respond to visual stimulation more than auditory stimulation.
Blinding	Blinding during group allocation is not relevant to this study because the primary comparisons were within subject. Furthermore in all paradigms, each subject was exposed to multiple conditions in rapid succession (1 or 10 sec trials, depending on the paradigm). This interleaved design controls for a variety of factors including potential influence by the experimenter.

Reporting for specific materials, systems and methods

We require information from authors about some types of materials, experimental systems and methods used in many studies. Here, indicate whether each material, system or method listed is relevant to your study. If you are not sure if a list item applies to your research, read the appropriate section before selecting a response.

Materials & experimental systems

n/a	Involved in the study
☒	☐ Antibodies
☒	☐ Eukaryotic cell lines
☒	☐ Palaeontology and archaeology
☒	☐ Animals and other organisms
☐	☒ Clinical data
☒	☐ Dual use research of concern
☒	☐ Plants

Methods

n/a	Involved in the study
☒	☐ ChIP-seq
☒	☐ Flow cytometry
☒	☐ MRI-based neuroimaging

Clinical data

Policy information about clinical studies

All manuscripts should comply with the ICMJE guidelines for publication of clinical research and a completed CONSORT checklist must be included with all submissions.

Clinical trial registration	NCT04188834
Study protocol	https://clinicaltrials.gov/ct2/show/NCT04188834
Data collection	Location of data collection: Epilepsy Monitoring Unit (EMU), Emory University Hospital.
Outcomes	Primary Outcome Measure:<br>Measure Title: Fold-change in Oscillatory Activity (Power Spectral Density) in Response to Exposure to Sensory Flicker: Comparing Mean Power Spectral Density at the Frequency of Flicker Being Presented Between Flicker and Baseline Periods<br>Measure Description: The power spectral density of the LFP will be measured across stimulus frequencies and modalities of sensory flicker stimuli in visual areas, auditory areas, hippocampus, and prefrontal cortex. To evaluate the effects of sensory flicker on brain activity in various brain regions, researchers compared the average increase in oscillatory neural activity of given recorded brain regions during sensory stimulation, among the total number of recording locations that showed a significant response to sensory stimulation compared to baseline. In participants in whom a condition was repeated across multiple experimental sessions. If a location showed a significant response in multiple sessions, the data point that showed the highest level of response was kept. The average fold-change increase in oscillatory activity, 25th and 75th percentiles, within a region of interest is reported.<br>Time Frame: During experiment session (up to 2 hours) during hospital admission (up to 2 weeks).<br><br>Secondary Outcome Measure:<br>Measure Title: Effect of Sensory Flicker on the Rate of Interictal Epileptiform Discharges (IEDs) Which Represent Pathological Activity Often Observed in Epilepsy<br>Measure Description: The change of of the sensory flicker effect will be evaluated by the comparison of the whole-brain rate of IEDs between sensory flicker stimulation and baseline (no stimulation).<br>Time Frame During experiment session (up to 2 hours) during hospital admission (up to 2 weeks).

Plants

Seed stocks	*Report on the source of all seed stocks or other plant material used. If applicable, state the seed stock centre and catalogue number. If plant specimens were collected from the field, describe the collection location, date and sampling procedures.*
Novel plant genotypes	*Describe the methods by which all novel plant genotypes were produced. This includes those generated by transgenic approaches, gene editing, chemical/radiation-based mutagenesis and hybridization. For transgenic lines, describe the transformation method, the number of independent lines analyzed and the generation upon which experiments were performed. For gene-edited lines, describe the editor used, the endogenous sequence targeted for editing, the targeting guide RNA sequence (if applicable) and how the editor was applied.*
Authentication	*Describe any authentication procedures for each seed stock used or novel genotype generated. Describe any experiments used to assess the effect of a mutation and, where applicable, how potential secondary effects (e.g. second site T-DNA insertions, mosiacism, off-target gene editing) were examined.*
